# Writing Alone or Together: Police Officers’ Collaborative Reports of an Incident

**DOI:** 10.1177/0093854818771721

**Published:** 2018-05-10

**Authors:** Annelies Vredeveldt, Linda Kesteloo, Peter J. van Koppen

**Affiliations:** VU University Amsterdam

**Keywords:** memory, police report, collaborative recall, memory conformity, retrieval strategy

## Abstract

After witnessing an incident, police officers may write their report collaboratively. We examined how collaboration influences the amount and accuracy of information in police reports. Eighty-six police officers participated, in pairs, in a live training scenario. Officers wrote a report about the incident, either with their partner or individually. Reports by two officers working together (collaborative performance) contained less information than reports by two officers working individually (nominal performance), with no difference in accuracy. After the first report, officers who had worked individually wrote a collaborative report. Police officers who recorded their own memories prior to collaboration included less incorrect information in the collaborative report than police officers who wrote a collaborative report immediately after the incident. Finally, content-focused retrieval strategies (acknowledge, repeat, rephrase, elaborate) during the officers’ discussion positively predicted the amount of information in collaborative reports. Practical recommendations for the police and suggestions for further research are provided.

Police officers witness a wide range of events about which they are required to write a report, such as criminal acts observed during stakeouts or patrols; conversations about illegal activity overheard via phone taps; observations of weapons, drugs, and other evidence during house searches; and descriptions of their own and suspects’ behavior during arrests. Those police reports can have a major impact in criminal cases (see, for example, S. Z. Fisher, 1993; Vredeveldt & van Koppen, 2016). Particularly in civil law systems, written police reports play a central role in legal proceedings. Incomplete and incorrect information in police reports can impede the investigation, prosecution, and adjudication of crimes. It is of paramount importance that the fact finder—judge or jury—can rely on police reports. Even though some studies address differences in memory performance between police and civilian witnesses (see Vredeveldt & van Koppen, 2016, for an overview), only a few studies address whether police reports are influenced by external factors in the same way as “regular” eyewitness statements. For example, we know that discussion with other witnesses can change what witnesses remember (e.g., Gabbert, Memon, & Allan, 2003; Paterson & Kemp, 2006; Vredeveldt, Hildebrandt, & van Koppen, 2016). In the present study, we examined whether discussion with another police officer affects their incident reports in the same way.

It is clear that collaboration affects cognitive performance. Researchers have discovered an effect called social loafing: when participants work together on a task, they often put in less individual effort (Latané, Williams, & Harkins, 1979). That may be due to a myriad of factors, such as that people believe their contribution will not make a difference to the group outcome or that they feel less responsible for the group’s outcome than for their own individual outcome (e.g., Harkins, 1987; Latané & Nida, 1981). Indeed, contrary to popular belief, brainstorming in a group actually has an adverse effect on creative thinking and productivity (Paulus, 2000; Taylor, Berry, & Block, 1958).

More specifically, collaboration affects recall performance. In collaborative recall studies (see Marion & Thorley, 2016, for an overview and meta-analysis), the performance of a group of individuals working together to recall (i.e., collaborative group) is compared to the pooled performance of the same number of individuals working individually (i.e., a nominal group). Most studies on collaborative recall have concerned the written recall of simple stimuli, although some recent studies have examined written recall of witnessed events as well (Bärthel, Wessel, Huntjens, & Verwoerd, 2017; Wessel, Zandstra, Hengeveld, & Moulds, 2015; Yaron-Antar & Nachson, 2006). A consistent finding is that collaborative groups remember significantly less on average than nominal groups, an effect called collaborative inhibition (Weldon & Bellinger, 1997). Importantly, collaborative inhibition does not seem to be due to social loafing (Weldon, Blair, & Huebsch, 2000). It may instead be due to individuals disrupting each other’s retrieval strategies, for example, by recalling the stimuli in a different order or by interrupting their partners’ contributions (see, for example, Barber, Harris, & Rajaram, 2015; Basden, Basden, Bryner, & Thomas, 1997). In previous studies on collaborative recall of witnessed incidents, collaborative inhibition has been observed for written reports (Bärthel et al., 2017; Wessel et al., 2015; Yaron-Antar & Nachson, 2006), but not for oral reports (i.e., eyewitness interviews; Vredeveldt, Groen, Ampt, & van Koppen, 2017; Vredeveldt et al., 2016).

The loss in recall quantity as a result of collaboration is typically offset by an increase in quality: collaborative groups make significantly fewer errors than nominal groups (e.g., Harris, Barnier, & Sutton, 2012; Hyman, Cardwell, & Roy, 2013; Ross, Spencer, Blatz, & Restorick, 2008). This error-pruning effect stands in sharp contrast to findings in the eyewitness literature, which show that witnesses often adopt each other’s errors (known as social contagion, see Roediger, Meade, & Bergman, 2001, or memory conformity, see Wright, Self, & Justice, 2000). These findings may seem contradictory, but are likely due to differences in methodology. In eyewitness studies, researchers are predominantly concerned with tracking the trajectory of errors that have been intentionally introduced by a confederate or by a co-participant who has seen a different version of the event. The overall amount of correct and incorrect information reported is usually not assessed. In collaborative recall studies, researchers are interested in assessing both correct recall and naturally introduced errors in a group of individuals recalling together, as compared to the same number of individuals recalling individually. The few studies that bridge the two literatures by examining collaborative recall in an eyewitness setting show that witnesses are more likely to prune each other’s errors than adopt each other’s errors. Error pruning in collaborative eyewitness recall is observed both for written reports (Bärthel et al., 2017; Wessel et al., 2015; Yaron-Antar & Nachson, 2006) and for oral interviews (Vredeveldt et al., 2016; 2017).

The effect of collaboration on recall performance is likely to depend on the relationship between partners. Transactive memory theory postulates that this relationship plays an important role in the success of a collaborative recall attempt (Wegner, 1987, 1995). When two or more individuals remember together, they will do well if individual group members have access to information that the other members do not have (differentiation) and also have shared knowledge of the type of information that other members have (integration). If those two requirements are met, the group as a whole should remember more than all of its individual members combined. In support of this theory, several studies suggest that partners who know each other well, and use their transactive memory system, remember more together than partners who do not know each other (Hollingshead, 1998; Johansson, Andersson, & Rönnberg, 2000; Wegner, Erber, & Raymond, 1991; but see Gould, Osborn, Krein, & Mortenson, 2002). Transactive memory theory also proposes that a successful transactive memory system takes time to develop (Tollefsen, 2006; Wegner, 1987). Based on that notion, one would predict that partners who have known each other for longer and have more frequent contact would remember better together. However, this prediction has not been borne out in previous studies, perhaps because the transactive memory system does not improve further once the partners have known each other for a relatively short amount of time (Vredeveldt et al., 2016; Wegner et al., 1991) or because a large amount of time spent together does not guarantee a good quality relationship (Barnier et al., 2014; Johansson, Andersson, & Rönnberg, 2005).

The effectiveness of collaboration also depends on the retrieval strategies used during collaboration. Some pairs help each other remember, whereas other pairs hurt each other’s recall (see e.g., Harris, Barnier, Sutton, & Keil, 2014; Harris, Keil, Sutton, Barnier, & McIlwain, 2011; Meade, Nokes, & Morrow, 2009). Vredeveldt and colleagues (2016; 2017) identified two distinct interaction styles during collaborative recall, each involving different retrieval strategies. Pairs of witnesses that adopt a content-focused interaction style are predominantly concerned with the content of what they are trying to remember together. They frequently acknowledge, repeat, rephrase, and elaborate upon each other’s contributions. In the research by Vredeveldt and colleagues, this interaction style was associated with an increased amount of information reported in eyewitness interviews, though it did not predict the accuracy of the information. Pairs of witnesses that adopt a process-focused interaction style, however, are predominantly concerned with the process of remembering together. They frequently refer to the relationship, explain their own statements, try to cue each other, check the accuracy of their own statements, and correct each other. This interaction style did not predict the amount or accuracy of information reported in eyewitness interviews.

Although studies on collaborative recall abound, few have focused on police officers specifically. Some studies have addressed the effects of conferring on police recall (for an overview, see Clark & Stephenson, 1999), but in these studies, collaborative performance was compared to individual performance rather than nominal (pooled) performance. Unsurprisingly, groups typically remember more information and make fewer errors than individuals. In an interesting recent study, Hope, Gabbert, and Fraser (2013) investigated how a discussion among four to six police officers about a staged crime influenced the officers’ subsequent individual written statements about that crime. Hope and colleagues found no significant effect of discussion on the overall amount or accuracy of information reported in individual statements. However, they did find evidence for error transmission: almost half of the errors mentioned by one officer during the discussion were subsequently reported by at least one of his team members in the subsequent individual report.

Another relevant issue to consider is whether police officers individually record their memories prior to collaboration or not. In one of the experimental conditions in the study by Hope and colleagues (2013), police officers first wrote their own individual report and then conferred with colleagues, after which they were allowed to adjust their report if they wished (59% of the officers chose to do so). Interestingly, the error transmission effect described above did not occur at all in that condition: when officers had first written down their own memories and then overheard errors during the discussion, none of these errors were subsequently incorporated in their adjusted reports. Thus, it seems that writing an individual report first protects against the social transmission of errors. In a similar vein, research on the Self-Administered Interview shows that participants who complete a comprehensive written recall prior to encountering misleading information, are less likely to incorporate that misleading information into their recall during a subsequent oral interview (Gabbert, Hope, Fisher, & Jamieson, 2012). That may be due to enhanced discrepancy detection (Hall, Loftus, & Tousignant, 1984; Tousignant, Hall, & Loftus, 1986). That is, external information is less likely to be incorporated into memory if the person has already exerted effort to recall the event, because there is a greater chance that he will notice a discrepancy between his memory and the information presented to him by someone else.

Individual recall prior to collaboration may not only affect the transmission of errors, but also the amount of information reported during the collaborative recall. Every time that an event is recalled, the memory is reactivated and the memory trace is reconsolidated (Alberini, 2005; Tronson & Taylor, 2007). Research shows that eyewitnesses are substantially more likely to retrieve information about a witnessed event if they have retrieved the same information on an earlier occasion (Burke, Heuer, & Reisberg, 1992; Shaw, Bjork, & Handal, 1995; Turtle & Yuille, 1994). The finding that the act of testing one’s memory can enhance later retrieval is known as the testing effect (Carrier & Pashler, 1992; Roediger & Karpicke, 2006). Moreover, during multiple retrieval attempts, people may use varying routes to access the information, which also results in increased memory output (Gilbert & Fisher, 2006; Schacter, Norman, & Koutstaal, 1998). This is the rationale behind the varied retrieval attempts featuring in the Cognitive Interview for witnesses (R. P. Fisher & Geiselman, 1992). Thus, there is abundant evidence in the memory literature that an initial recall attempt increases memory output during a second recall attempt. The role of collaboration in repeated recall is, however, less apparent. Some studies in the collaborative recall literature show that an individual recall opportunity prior to collaboration does not increase memory output during collaboration (i.e., it does not prevent collaborative inhibition; Blumen & Rajaram, 2008; Blumen, Young, & Rajaram, 2014; but see Congleton & Rajaram, 2011). In these studies, however, the experimental setup did not reflect what would happen in a police context. That is, study participants were not allowed to consult a copy of their individual recall attempt during the collaboration, whereas police officers would be able to consult their own individual reports while conferring with colleagues. This should lessen the disruption in individual retrieval strategies typically seen during collaboration (Basden et al., 1997) and is therefore expected to reduce collaborative inhibition.

In summary, when individuals write a report about a witnessed incident together (i.e., a collaborative group), they typically record fewer correct details but also make fewer errors than the same number of individuals working on their own (i.e., a nominal group; see, for example, Wessel et al., 2015). Whether collaborative inhibition and error pruning are observed, however, depends on the relationship between pair members and on the type of strategies they use while remembering together (e.g., Vredeveldt et al., 2016). A few studies have specifically addressed collaborative reports between police officers (see Clark & Stephenson, 1999), but most have compared collaborative performance to individual rather than nominal performance. Hope et al. (2013) found that conferring among groups of police officers prior to writing individual incident reports did not significantly affect the average number of correct or incorrect details in their reports, but did result in the transmission of specific errors mentioned during the discussion to team members’ subsequent individual reports. Importantly, however, the opportunity to write an individual report prior to conferring protected against the social transmission of errors.

## Present Study

The experimental design of the present study was quite different from the research conducted by Hope et al. (2013). Rather than examining the effect of collaboration on subsequent individual reports, we examined the effect of collaboration *during* the writing of the police report. That is, we compared the information provided by two police officers in one joint report (collaborative) with the information provided by two police officers in two individual reports (nominal). That comparison is highly relevant in the Dutch police context in which this research was conducted, because police officers in The Netherlands regularly submit a single police report signed by two or more officers. An informal survey in our professional network among police experts from Cyprus, Denmark, Finland, France, Iceland, Lithuania, Norway, Poland, South Africa, Sweden, the United Kingdom, and the United States revealed that this is quite rare. All of the surveyed experts informed us that police officers in their countries are legally required to submit individual police reports. Nevertheless, in practice, those guidelines are not always followed—sometimes, police officers do hand in a single report signed by multiple officers (this was noted by police experts from Belgium, France, Iceland, and Lithuania). Moreover, even when police officers submit individual reports, they may confer with colleagues before writing the report (this was noted by police experts in Cyprus and the United Kingdom). The common practice of conferring among police officers has also become apparent from survey data (Paterson & Kemp, 2005) and the issue has been raised in legal scholarly journals (e.g., Heaton-Armstrong, 1985; Stephenson, 1990; Wolchover, Heaton-Armstrong, Hope, & Gabbert, 2014) and police magazines (e.g., Baines, 1987; Pickover, 2009). Thus, the insights gained from the current study seem to be relevant for different legal systems.

In the present study, we sought to answer four research questions. The primary goal was to explore how collaboration during recall affects the content of police reports. Based on the collaborative recall literature (Marion & Thorley, 2016), we hypothesized that one collaborative report written by two police officers would contain less information than two individual reports (collaborative inhibition), but would be more accurate (error pruning).

Our second research question was how the recording of individual memories prior to collaboration would affect the content of the collaborative report. Pairs of police officers either wrote the collaborative report immediately after the incident or only after they had first written individual police reports about the incident. Based on previous findings (Gabbert et al., 2012; Hope et al., 2013), we hypothesized that police officers in the latter group would be less likely to incorporate incorrect information encountered during the discussion into their collaborative report. Furthermore, we expected that the initial individual retrieval attempt, in combination with the opportunity to consult individual reports while conferring, would increase the amount of information reported in the subsequent collaborative report (cf. Burke et al., 1992; Shaw et al., 1995; Turtle & Yuille, 1994).

Third, we examined the influence of the relationship between the police officers who were recalling together. Based on the transactive memory literature (e.g., Wegner et al., 1991), we predicted that police partners who knew each other before participating in our experiment would provide more information in their collaborative report than partners who did not know each other. Based on previous findings (e.g., Vredeveldt et al., 2016), we did not expect to observe an association between the duration or intensity of the relationship between police partners and the quality or quantity of their collaborative report.

Finally, we examined whether the police partners’ style of communicating with each other predicted the content of the collaborative report. Based on previous research on eyewitness interviews (Vredeveldt et al., 2016; 2017), we predicted that a content-focused interaction style—consisting of retrieval strategies of acknowledging, repeating, rephrasing, and elaborating upon each other’s contributions—would be associated with an increase in the amount of information reported, with no difference in the accuracy of the information.

## Method

### Participants and Design

Eighty-six police officers (75 males and 11 females) participated in the study. All participants were employed by the Dutch National Police Force but were stationed in different Regional Units throughout the country. Their age varied from 24 to 62 years (*M* = 41.15 years, *SD* = 11.02 years). Participants had been working for the police for 17.39 years on average (*SD* = 11.84, range = 2-43 years) and had on average 16.93 years of experience with writing police reports (*SD* = 11.87, range = 2-41 years). Officers indicated they wrote between 0 and 50 police reports per month, with an average of 10.88 per month (*SD* = 11.43).

All participants were randomly divided into pairs. Twelve pairs consisted of participants who did not know each other prior to participation and 30 pairs of participants who did know each other (one pair did not complete the questionnaire). The participants in the latter group had known each other for between 1.5 months and 30 years (*M* = 7.68 years, *SD* = 7.50 years) and saw each other regularly, with an average self-report rating of 4.18 (*SD* = 1.85) on a scale ranging from 1 (*seldom*) to 7 (*very often*).

All pairs were randomly assigned to experimental condition in a mixed design. The 23 pairs in the individual–collaborative (IC) condition first wrote an individual report and then a collaborative report. The 20 pairs in the collaborative–individual (CI) condition first wrote a collaborative report and then an individual report. Two pairs in the IC condition and two pairs in the CI condition were unable to finish their participation; they completed only the first police report. This resulted in 46 individual and 20 collaborative reports at Time 1 and 36 individual and 21 collaborative reports at Time 2. There were no significant differences between experimental conditions in terms of gender, age, years working for the police, years of experience writing police reports, or the number of police reports written per month (see Table 1). Similarly, there were no significant differences in terms of the number of pair members that had known each other beforehand, how long they had known each other, how frequently they saw each other, or how large the difference in years of experience was between pair members (see Table 1).

**Table 1:** Demographic Details of Participants in Both Experimental Conditions

Continuous variables	Individual–collaborative		Collaborative–individual		Difference statistics	
	*M*	*SD*	*M*	*SD*	*t*	*p*
Age	42.23	12.08	39.98	9.75	0.94	.348
Years of experience in the police force	19.08	12.74	15.50	10.58	1.41	.161
Years of experience writing police reports	18.06	13.05	15.66	10.39	0.93	.355
Police reports per month	12.26	12.58	9.37	9.96	1.13	.262
Relationship duration	7.24	8.95	3.34	3.63	1.87	.071^a^
Frequency of contact	2.70	2.51	3.19	2.48	0.62	.540
Event duration (min)	8.37	2.82	7.97	2.50	0.49	.630
Discussion duration (min)	37.57	16.98	39.42	16.35	0.35	.728
Categorical variables					χ^2^	*p*
Gender	40 males	6 females	35 males	5 females	0.01	.940
Gender composition of pair	18 same	5 mixed	16 same	4 mixed	0.02	.889
Knew partner beforehand?	15 yes	7 no	15 yes	5 no	0.24	.625

a This difference approached statistical significance, but this variable was significantly positively skewed (*Z* = 4.90) and leptokurtic (*Z* = 4.39) and showed significant heterogeneity of variance, *F*(1, 39) = 12.25, *p* = .001. Nonparametric tests revealed no significant difference between conditions, *U* = 177.00, *p* = .404.

### Materials

The incident about which police officers were asked to report concerned a live training exercise that was part of their continuing education program. In collaboration with police trainers, we developed a specific case training exercise for this research project, involving a suspicious man in a car. The police officers participated in the exercise in pairs. The training exercise was run by two police trainers: one who played the role of the police dispatcher providing information via a portable radio link (walkie-talkie) and one who played the role of the suspicious man in the car (the suspect).

At the start of the exercise, the trainer who later played the dispatcher provided each participant with a training gun, training pepper spray (containing water), and handcuffs. At least one of the officers in the pair also received a portable radio to communicate with the dispatcher. Participants were instructed to follow the instructions provided by the dispatcher and act upon that information as they saw fit. As the participant pair set off on the outdoor training grounds, they received the first message from the dispatcher: someone had reported a suspicious man in a car that had been parked in the same location for hours. The dispatcher explained where the car was spotted and the police pair walked toward that location. The dispatcher then asked the participants to provide the car’s license plate number, so that he could run it through the system. Once the participants had provided the license plate number, the dispatcher told them that the owner had a few outstanding traffic fines, which did not require an arrest. The police officers walked up to the suspect to chat with him.

A little while after the participants had engaged the suspect, the dispatcher corrected his earlier message: there was in fact a warrant out for the driver’s arrest to pay the traffic fines. Moreover, the suspect had been in a fight last week involving knives and baseball bats. Unbeknownst to the participants, we had also put a large carving knife in the car door’s side pocket. Now, the police participants’ goal was to arrest the suspect. The police trainer who played the role of the suspect had received instructions to struggle and resist arrest a little, but not use any physical violence. Once the police officers had arrested the suspect, the exercise was finished.

Although the script for the exercise was the same for all participating police pairs, its execution varied from case to case. Data collection was spread out over 10 days in three training locations in The Netherlands, with different cars and different police trainers who played the role of the dispatcher and the driver-suspect (with 11 different suspects in total). The progress of the training exercise also depended heavily on the decisions made by the police pair and their behavior. Some pairs arrested the suspect almost immediately, whereas other pairs engaged in a long conversation with the suspect before arresting him. Some pairs detected the knife and confiscated it, whereas other pairs did not detect the knife or did not act upon seeing it. The police trainer who played the suspect also reacted differently to police participants depending on their behavior: authoritative and physically confronting behavior tended to elicit more resistance from him (i.e., swearing and some physical struggle). The incident took between 3 and 16 min (*M* = 8.18 min, *SD* = 2.65 min), with no significant difference between conditions (see Table 1).

In sum, because of the realistic nature of the incident and the training goals of the police trainers, each case was different. To document what had happened during each case, we video-recorded each exercise with two cameras: one GoPro HERO action camera (attached to the chest of one of the participants) and one handheld camera (wielded by a researcher who followed the participant pairs during the entire exercise). Another researcher audio-recorded the information provided by the dispatcher via the portable radio link. All of these recordings were later used to code the information in the police reports provided by each specific police pair.

### Procedure

This research was incorporated in the continuing education training program for experienced operational police officers at large outdoor training grounds at one of three police training locations in The Netherlands (Almere, Leusden, and Elst). On each training day, groups of 10 to 30 officers arrived at the training location. Upon arrival, the police trainers informed them that researchers from a local university would be running a research study that day, in which the police officers could choose to participate in-between other training activities planned for the day (e.g., fitness tests, shooting exams). Police officers were informed that participation in the research would involve taking part in a case training exercise in pairs, followed by writing two police reports about the exercise. They were informed that the exercise would be video-recorded so that the researchers could check what happened during the exercise and that the video recordings would not be accessed by anyone outside of the research team and would not be used to evaluate their performance. Participants were informed that the goal of the study was to investigate different ways of writing police reports. Participants were not informed that the research concerned the role of collaboration between police officers, nor that they would be asked to write police reports in pairs. After being informed about the goals of the research, police officers were given the opportunity to ask questions. Those who participated signed an informed consent form. Participants were not financially compensated for their participation.

After taking part in the case training exercise described in the “Materials” section, police participants were accompanied on a 5-min walk to the building in which they would write their police reports. Participants were instructed not to talk to each other about the training exercise. For training purposes, however, they did receive some general feedback from the police trainer on how to handle situations like this. From an experimental point of view, this feedback was not ideal because, despite the fact that the trainer limited himself to general advice, the feedback may have affected how the participants remembered the exercise. From a practical point of view, however, the police trainers considered the immediate feedback necessary to ensure optimal benefits for the participating police officers.

Once the participants had arrived at the building, they were sat at a desk with a computer, either individually or together with their partner. They first received a two-page information sheet with instructions and tips on how to write a good police report, based on a Dutch manual for police officers (Huisman, Pranger, & Steenwinkel, 2007). Participants were allowed to keep the information sheet with them while writing their reports. Participants then wrote two police reports, one individually (each at their own computer) and one collaboratively (together at one computer), the order of which was determined by experimental condition, as explained in the “Participants and Design” section. Once each report had been completed, each participant was asked individually about their confidence in the report (i.e., “What percentage of the information you have provided do you believe to be correct?”).

The discussion between participants while writing the collaborative report was audio-recorded. Prior to writing the collaborative report, pair members were informed that they would together hand in one single report and would thus have to reach consensus on what to include in the report. Participants in the IC condition were allowed to consult a hard copy of their individual reports while writing their collaborative report.^1^ They were instructed not to copy and integrate the information from their individual reports verbatim, but rather use their individual reports to check that they had not forgotten anything.

At the end of the session, each participant provided demographic information (e.g., age and gender) and information about their employment and experience with writing police reports. They also answered questions about the relationship with their partner in the experiment (e.g., relationship duration and frequency of contact).

### Coding of Reports

Because each case was different, it was not possible to create a single coding scheme for what happened in the incident. Therefore, each police report was coded based on a general guidebook in combination with the video recordings of that particular case (GoPro and handheld camera), the audio recording of the dispatcher, demographic details of the suspect (e.g., age, height, weight, etc., provided by the suspect himself), and photos of the suspect, the car, and the surroundings. The general guidebook contained instructions on what constituted a single detail (e.g., hair color, hair length, and hair structure were all coded as separate details) and examples of what constituted subjective details (e.g., describing hair as “beautiful”).

All 123 written police reports were coded by two independent coders, blind to experimental condition. Each detail was coded as correct, incorrect, or subjective. In addition, the type of detail was coded: persons (e.g., the appearance and clothing of the suspect), actions by police officers (e.g., how they approached the suspect and whether they handcuffed him), actions by others (e.g., the behavior of the suspect and statements made by the dispatcher), objects (e.g., descriptions of the car and the knife), and surroundings (e.g., about the name of the street or a description of the parking area). To keep coding decisions as consistent as possible, the coding guidebook contained a list of predetermined rules. For example, estimates of the suspect’s age, height, and weight were coded as correct if they were within a range of 5 years younger or older, 5 cm shorter or taller, and 5 kg lighter or heavier than the demographic information provided by the suspect himself. Vague estimations such as “looked young” were coded as subjective details.

All reports were double-coded to determine interrater reliability. Interrater reliability was high for accuracy (κ = .86, *p* < .001; κ maximum = .95) and for type of detail (κ = .81, *p* < .001; κ maximum = .97). Coding disagreements were resolved by discussion.

### Coding of Discussions

The discussions between pair members while writing the police report were audio-recorded and transcribed. For one pair in the CI condition, the audio recording failed, so we analyzed 40 transcribed discussions in total. All transcriptions were coded for retrieval strategies by two independent coders. We used the coding scheme developed by Vredeveldt et al. (2016; see Table 2), with the addition of one extra coding category that we encountered relatively frequently in discussions between police officers: checking the accuracy of a particular detail. Interrater reliability was high for the coding of retrieval strategies (κ = .82, *p* < .001; κ maximum = .98). Coding disagreements were resolved by discussion.

**Table 2:** Retrieval Strategy Coding Categories With Descriptions and Examples

Strategy	Description and examples	*M*	*SD*
Successful cue	Cuing attempt (e.g., “What was his name again?”) that is followed by retrieval of information by the partner (e.g., “It was Jansen” or “Something starting with a J”).	8.93	4.85
Failed cue	Cuing attempt (e.g., “What was his name again?”) that is not followed by retrieval of information by the partner (e.g., “I don’t remember”).	2.98	1.90
Acknowledgment/confirmation	Indicating support for a partner’s statement, such as “Yes,” “Hm hm,” or “That’s right.”	33.85	16.82
Correction/disagreement	Correcting a partner’s statement (e.g., “No, it was Pietersen”) or questioning its accuracy (e.g., “I remember it differently”).	7.45	6.27
Elaboration	Building on a partner’s statement by providing additional information (e.g., the statement “dark T-shirt” is elaborated upon by the partner with “dark blue”).	50.00	22.03
Explanation	Explaining one’s own statement to the partner (e.g., “I remember his name was Tat because I remember thinking: what a strange name”).	2.32	2.69
Repetition	Repeating a partner’s statement verbatim.	3.10	2.64
Reformulation	Rephrasing a partner’s statement without changing the content (e.g., rephrasing “We made him sit” to “We brought him to the ground in a controlled manner”).	2.77	3.32
Renewed remembering	Indicating that a partner’s statement triggers a memory (e.g., “Now I remember it again” or “I had forgotten about that!”).	1.60	1.53
Relationship positive	Positive statement about the partner’s or the couple’s ability (e.g., “We’re a good team.” or “Good addition!”).	1.82	2.59
Relationship negative	Negative statement about the partner’s or the couple’s ability (e.g., “We didn’t pay much attention” or “I think we don’t remember this anymore”).	0.15	0.43
Role division/appoint expert	Dividing or organizing the retrieval task (e.g., “Shall I type?” or “You know more about cars than me”).	1.28	1.32
Checking accuracy	Checking with the partner whether a particular detail is correct (e.g., “He was wearing jeans, right?”).	15.18	7.83
Total number of strategies		131.42	57.16

*Note.* Means (*M*) and standard deviations (*SD*) of frequencies per collaborative interview (adapted from Vredeveldt, Hildebrandt, & van Koppen, 2016).

## Results

In dyadic designs, responses by individual pair members are typically not independent of each other and should not be treated as such in the analysis (Kenny, Kashy, & Cook, 2006). If there is any indication of nonindependence, as reflected in intraclass correlations significant at the liberal alpha level of .20 (Kenny et al., 2006; Myers, 1979), pair performance should be treated as the unit of analysis rather than individual performance. For the individual reports written prior to collaboration in the present study, the amount of information reported by the two pair members who had experienced the same event was significantly associated, *r* = .43, *p* = .034, whereas the percentage of information that was incorrect was not, *r* = .12, *p* = .578.^2^ Because there was a clear indication of nonindependence, we treated pair performance as the unit of analysis in all of our analyses.

Pair performance was calculated by adding up all nonredundant details (correct, incorrect, and subjective) reported by both pair members. Duplicate information was counted only once. We compared the performance of two police officers writing a single report together (collaborative performance) to the performance of two police officers writing their reports individually (nominal performance). Some of the variables were positively skewed, but square-root transformations resulted in normally distributed data for all variables. We conducted all analyses on both the original and the transformed variables, but the outcomes of the analyses did not differ. For ease of interpretation, we report only the analyses on the original variables for the amount of information reported and percentage incorrect.

### Initial Reports: Collaborative Versus Individual

The amount of information reported by the pair during the first recall attempt ranged from 52 to 230 details. Pairs that wrote the first report individually (IC condition) reported significantly more details (*M* = 126.78, *SD* = 42.34) than pairs that wrote the first report collaboratively (CI condition; *M* = 90.70, *SD* = 25.57), *t*(41) = 3.32; *p =* .002, *d* = −1.02; 95% CI = [–1.65, −0.37]. In line with our prediction of collaborative inhibition, collaborative pairs reported 28% less information than nominal pairs.

**Figure 1:**
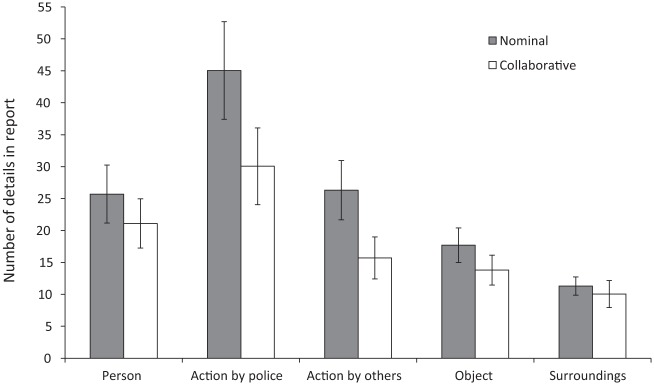
Average Number of Details Per Type of Detail in First Reports Written by Nominal Pairs and Collaborative Pairs *Note.* Error bars depict 95% confidence intervals.

To assess whether the conditions differed in the type of information police officers reported, we conducted an exploratory MANOVA with the total number of details about persons, actions by police officers, actions by others, objects, and surroundings as dependent variables (see Figure 1). There was a significant multivariate effect of condition, *F*(5, 37) = 2.98, *p* = .023, η^2^ = .29. The difference between conditions was significant for actions by the police, *F*(1, 41) = 8.73, *p* = .005, η^2^ = .18, and actions by others, *F*(1, 41) = 12.62, *p* < .001, η^2^ = .24. None of the other simple effects were significant at a Bonferroni-corrected α = .01 (all *p*s > .042, all η^2^s < .10).

Probably the most forensically relevant detail about the incident that the police officers could have mentioned in their report was the presence of the knife that was in the suspect’s car. We found that 13 out of 20 collaborative pairs mentioned the knife in their first report, compared to 13 out of 23 nominal pairs. The difference was not statistically significant, χ^2^(1) = 0.32; *p =* .571, Φ = .09. All pairs that had mentioned the knife in their first report also mentioned it in their second report. It should be noted that not all police pairs noticed the knife during the exercise, which explains why some of them did not mention it in their reports.

Percentage incorrect was calculated by dividing the number of incorrect details by the total number of details (correct, incorrect, and subjective). Of the information reported by the pair during the first recall attempt, 0% to 18% was incorrect. There was no significant difference between pairs that wrote the first report individually (*M* = 6.13%, *SD* = 2.72%) and pairs that wrote it collaboratively (*M* = 7.09%, *SD* = 4.49%), *t*(41) = 0.87; *p =* .392, *d* = 0.27; 95% CI = [–0.34, 0.87]. Thus, we found no support for our hypothesis that collaboration would result in error pruning.

### Collaborative Reports: With or Without Initial Individual Reports

The amount of information reported in the collaborative report ranged from 52 to 187 details. To examine whether recording individual memories prior to collaboration influenced the content of the collaborative report, we compared the collaborative report written immediately (CI condition) to the collaborative report written after writing an individual report (IC condition). The total number of details in the collaborative report did not differ significantly between the CI condition (*M* = 90.70, *SD* = 25.57) and the IC condition (*M* = 87.38, *SD* = 33.42), *t* (39) = −0.36; *p =* .724, *d* = −0.11; 95% CI = [–0.72, 0.50]. Thus, we did not find support for our prediction that writing an individual report prior to collaboration would increase the amount of information reported in the collaborative report.

To assess whether writing an individual report affected the amount of information about certain types of details but not others, we conducted an exploratory MANOVA on the total number of details about persons, actions by police officers, actions by others, objects, and surroundings. There was no significant multivariate effect of condition, *F*(5, 35) = 0.72, *p* = .612, η^2^ = .09, and none of the simple effects were significant at a Bonferroni-corrected α = .01 (all *p*s > .201, all η^2^s < .05).

The percentage of information in the collaborative report that was incorrect ranged from 0% to 18%. Percentage incorrect was significantly higher in the CI condition (*M* = 7.09%, *SD* = 4.49%) than in the IC condition (*M* = 3.85%, *SD* = 2.55%), *t* (39) = −2.87; *p =* .007, *d* = −0.90; 95% CI = [–1.52, −0.26]. Thus, we found support for our hypothesis that prior individual reporting would reduce the percentage of incorrect information in the collaborative report.

### Relationship Between Partners

Of the police pairs that wrote a collaborative report, 30 pairs consisted of partners who knew each other prior to participation and 11 pairs of partners who did not know each other. Note that the sample size in the latter group was rather small to draw any firm conclusions. Nevertheless, we present some exploratory data below. Contrary to our prediction, partners who knew each other provided significantly fewer details in their collaborative report (*M* = 81.43, *SD* = 23.22) than partners who did not know each other (*M* = 109.64, *SD* = 35.84), *t* (39) = 2.96; *p =* .005, *d* = −1.04; 95% CI = [–1.77, −0.31]. Among the 30 pairs of partners who knew each other, we also examined the correlation between the duration of the partners’ relationship and the number of details in their collaborative report. We found no significant correlation, *r*(29) = –.30, *p* = .113, *r*^2^ = .09. Similarly, there was no significant correlation between the frequency of contact between the partners, as rated on a 7-point scale ranging from “seldom” to “very often,” and the number of details in their collaborative report, *r* (28) = .14, *p* = .473, *r*^2^ = .02.

We found no statistically significant difference in the percentage of incorrect information in the collaborative report between partners who knew each other prior to participation (*N* = 30, *M* = 4.93%, *SD* = 3.87%) and partners who did not know each other (*N* = 11, *M* = 6.79%, *SD* = 3.96%), *t* (39) = 1.36; *p =* .183, *d* = −0.48; 95% CI = [–1.17, 0.22]. Among the 30 pairs of partners who knew each other, we found no significant correlation between relationship duration and percentage of incorrect information in the collaborative report, *r*(29) = –.07, *p* = .728, *r*^2^ = .00. Similarly, we found no significant correlation between frequency of contact between the partners and percentage of incorrect information in the collaborative report, *r* (28) = .19, *p* = .326, *r*^2^ = .04.

### Retrieval Strategies

On average, police pairs took 38.45 min (*SD* = 16.49 min) to complete the collaborative report, with no significant difference between conditions (see Table 1). In that time, pairs uttered about 369 statements on average, of which 36% reflected one of the retrieval strategy categories shown in Table 2. The two most commonly used strategies were to acknowledge each other’s contributions and to elaborate upon each other’s statements. Prior to analysis, all retrieval strategies were square-root transformed to counter positive skew and leptokurtosis. Negative references to the relationship occurred so infrequently that the variable could not be normalized. It was therefore excluded from the analyses. Based on previous research (Vredeveldt et al., 2016; 2017), the remaining strategies in Table 2 can be categorized into two distinct interaction styles: content-focused interaction (acknowledge, repeat, rephrase, elaborate) and process-focused interaction (successful cue, failed cue, correction, explanation, renewed remembering, relationship positive, role division, checking accuracy).

A linear regression analysis was conducted to examine whether the two types of interaction predicted the amount of information in collaborative police reports. The strategies associated with content-focused interaction, which were hypothesized to positively predict the amount of information, were entered into the regression model first. The strategies associated with process-focused interaction, which were hypothesized to have no effect, were entered next. The model with content-focused strategies explained a significant proportion of the variance in the amount of information reported (*R*^2^ = .44; see Table 3). The number of elaborations in the discussion was a significant positive predictor of amount reported, *β* = .74, *t*(37) = 3.12, *p* = .004, but the number of reformulations was a significant negative predictor of amount reported, *β* = –.34, *t*(37) = −2.07, *p* = .046. The other content-focused strategies were not significant predictors. Adding process-focused strategies to the model did not significantly increase the proportion of variance explained.

**Table 3:** Results of Multiple Linear Regression Analysis With Retrieval Strategies as Predictors of the Amount of Information Reported and the Percentage of Details That Were Incorrect

	Amount reported			Percentage incorrect		
Predictor	*B*	*SE B*	*β*	*B*	*SE B*	*β*
Step 1						
Constant	4.71	0.95		1.11	0.72	
Acknowledgment	0.12	0.20	.12	0.11	0.15	.18
Repetition	−0.06	0.31	−.03	0.01	0.23	.01
Reformulation	−0.49	0.24	−.34*	−0.06	0.18	−.07
Elaboration	0.69	0.22	.74**	0.07	0.17	.12
Step 2						
Constant	5.90	1.06		0.44	0.86	
Acknowledgment	0.05	0.24	.05	0.06	0.19	.10
Repetition	0.18	0.34	.10	−0.21	0.28	−.19
Reformulation	−0.47	0.25	−.33	−0.12	0.20	−.14
Elaboration	0.91	0.25	.98**	0.17	0.20	.30
Successful cue	−0.05	0.26	−.03	0.09	0.21	.11
Failed cue	0.11	0.42	.05	0.25	0.34	.17
Correction	−0.30	0.28	−.20	−0.18	0.23	−.20
Explanation	0.42	0.32	.25	−0.01	0.26	−.01
Renewed remembering	0.26	0.35	.13	−0.16	0.29	−.14
Relationship positive	0.22	0.22	.14	0.42	0.17	.47*
Role division	−0.24	0.30	−.12	−0.30	0.25	−.25
Checking accuracy	−0.75	0.27	−.52*	0.14	0.22	.16

*Note*. Content-focused strategies were entered in Step 1 and process-focused strategies in Step 2. All variables were square-root transformed prior to analysis to counter positive skew and leptokurtosis. For the amount reported, Step 1: *R*^2^ = .44, *F*(4, 34) = 6.61, *p* < .001; Step 2: Δ*R*^2^ = .21, *F*(8, 26) = 1.99, *p* = .088. For percentage incorrect, Step 1: *R*^2^ = .07, *F*(4, 34) = 0.60, *p* = .663; Step 2: Δ*R*^2^ = .28, *F*(8, 26) = 1.38, *p* = .252.

* *p* < .05. **p < .01.

Another linear regression assessed whether the two types of interaction styles predicted the percentage of incorrect information in the report. The model with only content-focused strategies did not explain a significant proportion of the variance (see Table 3), and adding process-focused strategies did not significantly increase the proportion of variance explained. Thus, neither type of interaction style predicted the percentage of inaccuracies in the collaborative report.

## Discussion

We explored the effect of collaboration between two police officers on the amount and accuracy of information in their police reports. First, as predicted, we found that two police officers who wrote their first police report together reported significantly less nonredundant information than two police officers who each wrote an individual police report. Collaborative inhibition was most pronounced for information about actions by the police officers and actions by others. We did not, however, find support for our error-pruning hypothesis: there was no significant difference between conditions in the percentage of information in the first police report that was incorrect. Second, our hypothesis that writing an individual report prior to collaboration would increase the amount and accuracy of information in the collaborative report was only partly supported: we did not observe the expected benefit for the amount of information reported, but we did find an increase in accuracy. Third, for the role of relationship in collaboration, the findings were opposite to what we expected: partners who knew each other beforehand reported significantly less information in their collaborative report than partners who did not know each other. Relationship duration and frequency of contact between acquainted partners was not related to the amount or accuracy of information in the collaborative reports. Finally, police partners who used content-focused strategies during the writing of the collaborative report provided significantly more information in that report.

The observed collaborative inhibition effect is in line with previous studies on written reports of witnessed incidents (Bärthel et al., 2017; Wessel et al., 2015; Yaron-Antar & Nachson, 2006). Two heads apart know more than two heads together, probably because individuals have different, idiosyncratic ways of remembering information that interfere with each other (Barber et al., 2015; Basden et al., 1997). Interestingly, the robust collaborative inhibition effect is abolished in semi-structured oral interviews with eyewitnesses (Vredeveldt et al., 2016; 2017), perhaps because individual retrieval strategies do not play such an important role in eyewitness interviews. After all, the police interviewer’s questions provide retrieval cues to witnesses, which prevent them from forgetting to mention important topics. Alternatively, the interviewer’s questions may disrupt retrieval strategies for nominal pairs just as much as for collaborative pairs.

Surprisingly, the average percentage of incorrect information in the collaborative report (7%) did not differ significantly from the average percentage of incorrect information in the two individual reports (6%). We can only speculate about the reasons for the absence of an error-pruning effect in the current study, whereas it has been consistently observed in previous studies on written reports of witnessed incidents (Bärthel et al., 2017; Wessel et al., 2015; Yaron-Antar & Nachson, 2006). Perhaps it can be explained by a police culture in which questioning each other in general, and each other’s statements in particular, is not encouraged. During his 10 years as a detective in the Dutch police, Princen (2015) observed a culture in which contradicting or correcting each other is not appreciated. This culture is not unique to the Dutch police (see, for example, Chan, 1996; Crank, 2014; Goldsmith, 1990). In many situations that police officers face on a daily basis, there are good reasons for the reluctance to question or contradict each other. When talking to an aggressive citizen or arresting a suspect, it is wise to present a united front and not undermine each other’s authority. Goldsmith (1990) notes that solidarity between police officers “offers its members reassurance that the other officers will ‘pull their weight’ in police work, that they will defend, back up and assist their colleagues when confronted by external threats, and that they will maintain secrecy in the face of external investigations” (pp. 93-94).

Of course, this sense of solidarity may be less beneficial when officers are writing a police report together, when accuracy is of paramount importance.

In line with previous studies (Burke et al., 1992; Shaw et al., 1995; Turtle & Yuille, 1994), we predicted that an initial opportunity to recall the event would help officers report more information in their subsequent report. The experimental designs of those previous studies do not map perfectly onto the current design, however, because the second recall in our IC condition was collaborative rather than individual. Some previous studies on combinations of individual and collaborative recall attempts, using simple stimuli as the to-be-remembered material, have found that an initial individual recall attempt does not prevent collaborative inhibition during subsequent collaborative recall (Blumen & Rajaram, 2008; Blumen et al., 2014; but see Congleton & Rajaram, 2011). Nonetheless, we expected that officers in our study who had recalled the event individually first would remember more during collaborative recall than officers who had not yet recalled the event, because the former were able to consult their own individual reports during the discussion, which should reduce the retrieval strategy disruption typically observed in collaborative recall (Basden et al., 1997). However, this is not what we found. Collaborative reports in both conditions contained an equivalent amount of information. Thus, it seems that even the opportunity to recall the event individually first, and consult individual reports during the discussion, was unable to attenuate the robust effect of collaborative inhibition. However, this analysis was limited by the fact that collaboration was confounded with repeated recall (i.e., we compared a collaborative report that constituted the officers’ first recall attempt, in the CI condition, with a collaborative report that constituted the officers’ second recall attempt, in the IC condition). To tease apart the effects of collaboration and repeated recall, respectively, future studies should adopt a full crossover design by adding a condition with two collaborative reports (CC) and a condition with two individual reports (II).

Writing an individual report prior to collaboration did, however, reduce the percentage of incorrect information in the collaborative report. This mirrors Hope et al.’s (2013) findings that the social transmission of errors did not occur when police officers wrote their report individually first. It is also in line with previous findings that an initial free recall opportunity reduces susceptibility to misinformation (Gabbert et al., 2012; Geiselman, Fisher, Cohen, Holland, & Surtes, 1986; Memon, Zaragoza, Clifford, & Kidd, 2010 but see Wilford, Chan, & Tuhn, 2014). These findings may be explained by the discrepancy detection principle (Hall et al., 1984; Tousignant et al., 1986). If a police officer has not yet thought about the color of the suspect’s jacket, for example, and hears his colleague say that it was red, he may simply go along with that statement rather than examine it critically. If, however, the police officer has already written in his own individual report that the suspect was wearing a black jacket, he is more likely to notice the discrepancy between his memory and his colleague’s memory. If he notices that discrepancy, he may feel obliged to address it. After all, by signing the report, the police officer declares that the provided information is, to the best of his knowledge, accurate. When officers discuss the discrepancy, inaccurate memories are more likely to be changed than accurate memories (Vredeveldt et al., 2017; Wright & Villalba, 2012), which should result in a more accurate police report.

Surprisingly, police partners who did not know each other before participating provided significantly more information in their collaborative report than partners who did know each other. One explanation for this unexpected finding might be that partners who do not know each other feel that they have to prove themselves to their partner and therefore put more effort into remembering the incident. Because only 11 pairs in the present study were unacquainted partners, however, we cannot draw any firm conclusions regarding the difference between acquainted and unacquainted partners. Future research should investigate the role of partners’ prior acquaintance in collaborative report writing more systematically. For the 30 pairs of police partners who knew each other, we observed no significant associations between collaborative recall performance and relationship duration or frequency of contact. This is not surprising in light of previous findings which suggest that the minimum amount of time spent together to develop a transactive memory system is probably shorter than the average 8-year relationship between acquainted partners in the present study (cf. Vredeveldt et al., 2016; Wegner et al., 1991). When it comes to remembering together, it is probably not the amount of time spent together that counts, but rather the quality of the relationship (Barnier et al., 2014; Johansson et al., 2005) and the partners’ interaction style (Vredeveldt et al., 2016; 2017).

Our findings regarding the partners’ interaction style were generally in line with previous findings. Just like studies on eyewitness interviews (Vredeveldt et al., 2016; 2017), we found that the use of content-focused strategies during the discussion was associated with more information in collaborative reports. Interestingly, we replicated this finding even though participants in the present study provided a written report rather than an oral report. The observed number of relevant elaborations during the discussion was the most important positive predictor of the amount of information reported, which is again consistent with the above-mentioned studies on collaborative eyewitness interviews and also with findings on autobiographical recall by older married couples (Harris et al., 2011) and recall of flight scenarios by aviation experts (Meade et al., 2009). The only finding with regard to retrieval strategies that was not in line with previous research was that reformulations were negatively associated with the amount of information in the collaborative report. This is surprising because reformulating the partner’s statement is a feature of the content-focused interaction style, which generally predicts an increase in reported information. Anecdotal observations provided one potential explanation for this unexpected finding: In the context of writing a police report, reformulations may be disruptive because they focus the attention on how to phrase something in a formal manner, rather than on recalling additional information. In the context of oral recall of events, in contrast, reformulations seem to be signs of active listening that help individuals to carefully consider their partner’s response and elaborate with relevant new information.

One limitation of the present study was the lack of control over what happened during the live event. Each police pair handled the situation differently, resulting in events that varied widely in both nature and duration. Because we randomly assigned police officers to experimental conditions, this variation should not have biased the results toward one particular condition, but one can never be certain that a confound did not occur by mere chance. For the variables that we were able to check (e.g., demographic information, relationship between pair members, event duration, discussion duration), there was no indication that this was the case, but the groups still may have differed on some unknown variable (e.g., the events in one condition may coincidentally have been more salient). In a similar vein, we had only minimal control over what happened immediately following the event. For training purposes, police officers received some feedback from the police trainers prior to writing their incident reports. Ideally, we would have preferred for this feedback to have been given after the incident reports had been written, but the police trainers insisted on providing immediate feedback. They did agree, however, to limit themselves to general advice on how to handle this kind of situation, without mentioning any details on the specific case. Future research should eliminate this feedback altogether, if practically feasible.

Another limitation of the present study was that police officers were aware that the researchers would be analyzing their police reports. Participants may therefore have put more effort into writing their reports than they would in normal everyday practice (i.e., the “Hawthorne effect,” cf. Wickström & Bendix, 2000). This limitation does not affect the main outcome of the experiment, as officers in both experimental conditions were aware that their reports would be analyzed, but it does mean that the quantity and quality of police reports in this study may not be reflective of police reports in the real world. Indeed, the quality of police reports in the real world has been identified as a major area for improvement, because the reports are often incomplete or inaccurate (see e.g., Dutch Ministry of Security and Justice, 2015; Her Majesty’s Chief Inspector of the Crown Prosecution Service, 2016).

We would like to highlight two important outstanding questions on the practice of collaborative report writing. First, we are currently unaware of how frequently police officers in The Netherlands (or in other countries) write police reports collaboratively and whether that frequency depends on the type or severity of the case. During the present study, we asked various officials in the Dutch police, but nobody seemed to have an answer. Future studies should survey a large sample of police officers to examine the scale of collaborative report writing in various types of criminal cases. Second, when police officers submit a joint report, we do not know how they collaborate to arrive at that report. Do they sit down behind the computer together and discuss the incident while writing the report (as police participants did in the current study)? Does one officer write the first version of the report and the other revise and add to it later? Or does one officer write the report and the other merely sign it? When we posed these questions to police officials, their general answer seemed to be that it depends on the particular police officers involved. Future research should investigate how collaboration takes shape in practice, and moreover, how different forms of collaboration affect the amount and accuracy of information in the police report.

In conclusion, the information provided in police reports is paramount for the successful prosecution of criminal cases and the conviction of offenders. The present findings suggest that one way to improve the completeness of police reports would be to instruct police officers to write their reports individually, rather than collaboratively.

## Notes

1. We chose to do this because one goal of this study was to identify the best procedure for writing police reports. Allowing participants to consult their own individual reports could potentially mitigate the disruption of individual retrieval strategies that typically occurs during collaborative remembering, thereby reducing collaborative inhibition. Participants in the collaborative–individual (CI) condition were not allowed to consult their collaborative report while writing the individual report, because initial testing showed that they then simply copied the collaborative report (which did not happen in the individual–collaborative (IC) condition, as participants had to integrate their memories into one report).

2. Note that we computed intraclass correlations only for the IC condition, because the individual reports written in the CI condition were not assessed in our analyses. Individual reports written after collaboration (i.e., in the CI condition) were even more strongly correlated (amount of information: *r* = .75, *p* < .001; percentage incorrect: *r* = .65, *p* = .001).
